# Successful Open Reduction of Post-traumatic Calcaneocuboid Dislocation: A Case Report

**DOI:** 10.7759/cureus.91341

**Published:** 2025-08-31

**Authors:** Xchel Iván Campuzano-Rodríguez, Luis M Canizalez-Tripp, Jorge A Fernández-Kerber, David E Tanimoto-Abad, José Luis Silva-Hernández

**Affiliations:** 1 Orthopedic Surgery, Hospital General de Zona No. 7 IMSS, Monclova, MEX

**Keywords:** calcaneocuboid, calcaneus, dislocation, kirschner wires, midfoot

## Abstract

Calcaneocuboid dislocation is an exceptionally rare midfoot injury, typically resulting from high-energy trauma and often underdiagnosed due to subtle radiographic findings and the joint’s intrinsic stability. We report a case of a 15-year-old male who sustained an isolated calcaneocuboid dislocation following a motorcycle accident. Initial radiographs revealed incongruity of the lateral midfoot without associated fractures. Surgical management involved open reduction and temporary fixation with Kirschner wires to address intra-operative soft tissue interposition and restore joint congruity. Post-operative recovery was favorable, with complete wound healing, maintained reduction, and restoration of functional mobility at follow-up. This case underscores the importance of early recognition, precise imaging, and tailored surgical intervention to preserve lateral column integrity and optimize outcomes in adolescent patients with rare midfoot dislocations.

## Introduction

Dislocations and fractures of the midfoot are poorly known, with Chopart injuries involving the calcaneocuboid joint estimated at 2.2 per 100,000 person-years, a frequency attributed to the joint’s intrinsic stability provided by its saddle-shaped architecture, robust ligamentous support, and the proximity of the peroneus longus tendon [1,2]. Most cases result from high-energy trauma and are usually associated with complex Chopart injuries, while isolated calcaneocuboid involvement often escapes early detection [3]. Delayed or missed diagnosis is frequent, as standard radiographs may fail to demonstrate subtle joint incongruity. Advanced imaging can aid in detection; however, clinical criteria for early recognition remain undefined [4]. Surgical management is essential to restore lateral column alignment, prevent joint instability, and preserve load transmission across the hindfoot [5]. Disruption of this articulation can lead to lateral column shortening, progressive deformity, and post-traumatic arthritis if not anatomically reduced and stabilized. Open reduction and temporary Kirschner wire fixation have been proposed to restore joint congruity and minimize long-term sequelae [6]. This case highlights the diagnostic and surgical challenges of lateral midfoot dislocations in the adolescent population.

## Case presentation

A 15-year-old male student with no known chronic medical conditions reported active tobacco use and occasional alcohol consumption. He sustained high-energy trauma while operating a motorcycle at high speed. Upon attempting to brake, he lost control and collided head-on with a curb approximately 50 cm in height. During the impact, his left foot became trapped between the motorcycle and the curb, resulting in acute lateral midfoot pain and visible bleeding from a dorsal wound.

**Figure 1:**
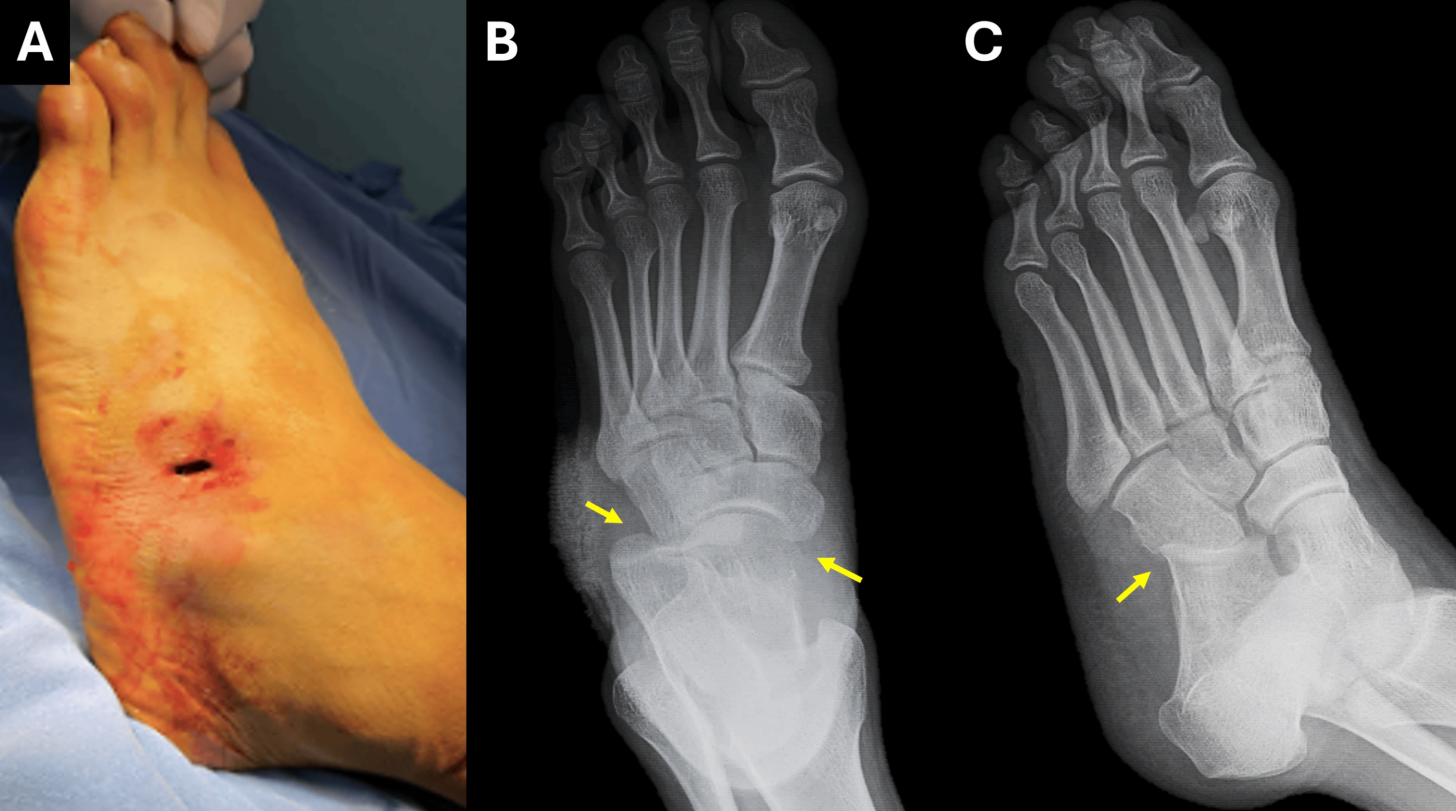
Clinical and radiographic findings of isolated calcaneocuboid dislocation. (A) Intra-operative clinical image of the left foot showing a 1 cm dorsal laceration over the lateral midfoot, located at the level of the fifth metatarsal, with clean margins and no visible contamination, consistent with high-energy traumatic injury. (B) Pre-operative anteroposterior radiograph demonstrating lateral displacement and articular incongruity at the calcaneocuboid joint (yellow arrows), without evidence of associated fractures. (C) Oblique view confirming isolated dislocation of the calcaneocuboid joint, with preserved alignment of adjacent joints and no radiographic signs of bony fragmentation, supporting the diagnosis and facilitating surgical planning (yellow arrow).

He was brought to the emergency department for evaluation. On arrival, he was alert, oriented, and hemodynamically stable. Physical examination of the left lower extremity revealed a 1 cm dorsal laceration over the fifth metatarsal region, with regular margins and no signs of contamination (Figure 1A). There was marked tenderness over the lateral midfoot. Ankle and toe range of motion was preserved and painless. Capillary refill was intact, and no neurovascular deficits were identified. Plain radiographs of the left foot (anteroposterior and oblique views) revealed lateral displacement and articular incongruity of the calcaneocuboid joint, without associated fractures (Figures 1B, 1C). The injury was diagnosed as an isolated calcaneocuboid dislocation, classified as AO/OTA type 80D2(5) and type V according to the Main and Jowett classification system.

**Figure 2:**
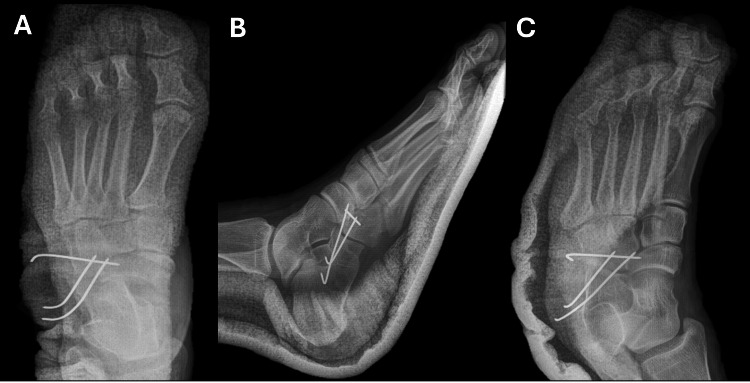
Post-operative radiographs following open reduction and internal fixation of isolated calcaneocuboid dislocation. (A) Anteroposterior radiograph of the left foot showing restoration of calcaneocuboid joint congruity and stable fixation using two 2.0 mm Kirschner wires across the calcaneocuboid joint and one across the cuboid-navicular joint. (B) Lateral view demonstrating proper depth and orientation of the Kirschner wires within the lateral column, confirming anatomical realignment of the hindfoot and midfoot articulations. (C) Oblique view illustrating maintenance of joint reduction and stable percutaneous fixation, without evidence of hardware migration or iatrogenic fracture.

Given the joint instability and failed closed reduction, surgical intervention was indicated. Under epidural anesthesia, the patient was placed in the supine position, and a sterile field was established. A 2 cm anterolateral incision was made over the calcaneocuboid joint under fluoroscopic guidance. Dissection proceeded through anatomical planes, with electrocautery used for hemostasis. Senn-Miller retractors were used to expose the joint space, revealing interposed soft tissue within the dislocated articulation. Anatomical reduction was achieved using Weber clamps and confirmed fluoroscopically. Fixation was performed using one 2.0 mm Kirschner wire across the cuboid-navicular joint and two 2.0 mm wires across the calcaneocuboid joint (Figures 2A-2C). The joint was irrigated thoroughly, and the wound was closed in anatomical layers. A posterior splint was applied, and intra-operative blood loss was estimated at 150 mL.

The patient remained under close post-operative observation. Clinical evaluation confirmed proper wound healing, absence of infection, and preserved range of motion of the ankle and toes. The Kirschner wires were removed without complication during outpatient follow-up, with no signs of joint instability or neurovascular impairment. Functional recovery was progressive, allowing a gradual transition to weight-bearing. Given the favorable clinical course, orthopedic follow-up was concluded without further intervention.

## Discussion

Isolated dislocation of the calcaneocuboid joint represents a rare entity within midfoot trauma, accounting for less than 5% of injuries in this anatomical region and typically arising from high-energy mechanisms [7]. Its intrinsic stability is conferred by the saddle-shaped articular surface, robust capsuloligamentous structures, and dynamic support from the peroneus longus tendon, making isolated dislocations uncommon in the absence of associated fractures [8,9].

While midfoot dislocations more commonly involve the tarsometatarsal (Lisfranc) or talonavicular joints, calcaneocuboid dislocations remain infrequently reported and frequently underrecognized [10]. These injuries are usually sustained during forceful abduction or inversion events, such as those seen in motorcycle accidents or crush mechanisms, leading to disruption of the lateral column of the foot [11].

Timely recognition is essential to prevent long-term sequelae, such as chronic pain, post-traumatic arthrosis, or collapse of the lateral longitudinal arch. However, diagnosis can be challenging due to the rarity of the lesion and the often subtle findings on plain radiographs. Although computed tomography (CT) is the preferred modality for detecting joint malalignment and excluding occult fractures in complex or equivocal cases, in the present case, standard radiographs were sufficient to identify joint displacement and rule out bony involvement, allowing for a focused and timely surgical approach [10,12]. This highlights the importance of maintaining a high index of suspicion when evaluating midfoot pain following high-energy trauma and tailoring imaging strategies to the clinical context [13].

Closed reduction remains the initial therapeutic maneuver; however, soft tissue interposition and capsuloligamentous entrapment frequently preclude anatomical reduction [11,14]. In our patient, open reduction was required due to the presence of obstructive soft tissue structures that impaired congruent joint alignment. Stabilization was achieved using 2.0 mm Kirschner wires spanning both the calcaneocuboid and cuboid-navicular joints, a method supported in previous reports for its simplicity, minimal invasiveness, and facilitation of early hardware removal without compromising joint surfaces [15].

Post-operatively, the patient demonstrated favorable outcomes, with preservation of joint mobility, absence of infection, and successful progression through staged weight-bearing. Radiographic consolidation and restoration of foot biomechanics were achieved within the expected timeframe, consistent with published outcomes for early surgical intervention and structured rehabilitation [9,16]. These results underscore the importance of early anatomical realignment, rigid temporary fixation, and close post-operative monitoring to preserve midfoot integrity.

These findings align with the principles highlighted by Pérez et al., who emphasize that successful management of midfoot injuries requires three core elements as follows: (a) anatomical restoration of both medial and lateral columns, (b) reestablishment of joint congruity, and (c) temporary stabilization to facilitate soft tissue healing [17]. The present case contributes to the limited literature on calcaneocuboid dislocations in adolescents, demonstrating that even in the absence of CT imaging, early clinical suspicion, targeted radiographic assessment, prompt open reduction, and minimally invasive fixation can lead to excellent functional recovery, even in the context of high-energy trauma.

## Conclusions

This case illustrates that isolated calcaneocuboid dislocations, although rare, can be successfully managed with timely diagnosis, open reduction, and temporary fixation. Anatomical restoration of the lateral column is essential to prevent chronic instability, deformity, and degenerative sequelae. The surgical approach must be tailored to address soft tissue interposition and ensure joint congruity. While the biomechanical demands of the midfoot are well established, few reports detail the management of these injuries in skeletally immature patients. Continued documentation of such cases is critical to refining therapeutic strategies and supporting evidence-based care for complex midfoot trauma in adolescent populations.

## References

[1] Ponkilainen VT, Laine HJ, Mäenpää HM, Mattila VM, Haapasalo HH (2019). Incidence and characteristics of midfoot injuries. Foot Ankle Int.

[2] Moracia-Ochagavía I, Rodríguez-Merchán EC (2019). Lisfranc fracture-dislocations: current management. EFORT Open Rev.

[3] Kummer A, Crevoisier X, Eudier A (2020). Calcaneocuboid and naviculocuneiform dislocation: an unusual injury of the midfoot. Case Rep Orthop.

[4] Rammelt S, Marx C, Swords G, Swords M (2021). Recognition, treatment, and outcome of calcaneal fracture-dislocation. Foot Ankle Int.

[5] Kamakura F, Yasuda G, Ishigaki Y, Goto S (2023). An uncommon midfoot injury, naviculocuneiform and calcaneocuboid fracture dislocation: a case report. J Surg Case Rep.

[6] Vosoughi AR, Vallier HA (2021). Closed pantalar dislocations: characteristics, treatment approaches, and outcomes. J Am Acad Orthop Surg.

[7] Dhole KP, Bandebuche AR, Marathe NA, Date S, Raj A (2020). An unusual midfoot dislocation involving naviculocuneiform and calcaneocuboid joint following low-energy injury: a case report. J Orthop Case Rep.

[8] Yıldırım Y, Ergün S, Akgülle AH, Cansü E (2014). Calcaneocuboid joint dislocation: a case report. J Am Podiatr Med Assoc.

[9] Punwar S, Madhav R (2007). Dislocation of the calcaneocuboid joint presenting as lateral instability of the ankle. J Bone Joint Surg Br.

[10] Alberta FG, Aronow MS, Barrero M, Diaz-Doran V, Sullivan RJ, Adams DJ (2005). Ligamentous Lisfranc joint injuries: a biomechanical comparison of dorsal plate and transarticular screw fixation. Foot Ankle Int.

[11] Wong KP, Tang ZH, Tan GM (2020). Combined open calcaneocuboid, naviculocuneiform and subtalar dislocation: a case report and literature review. Biomedicine (Taipei).

[12] Alhadhoud MA, Alsiri NF, Mohammad DA, Ibrahim A, Aboubakr MK, Abdulghany M, Fathy A (2022). Open fracture dislocation of the calcaneocuboid and naviculocuneiform joints: a case report. Trauma Case Rep.

[13] Vosoughi AR, Akbarzadeh A, Zakaee A (2022). Closed isolated anterolateral calcaneal dislocation: a case report. BMC Musculoskelet Disord.

[14] Swords MP, Schramski M, Switzer K, Nemec S (2008). Chopart fractures and dislocations. Foot Ankle Clin.

[15] Ammari Sánchez-Villanueva F, Romero Sánchez M, García Guirao A, Martínez Sañudo B. Extraña luxación de mediopie: Disrupción de articulaciones escafo-cuneana y calcáneo-cuboidea, una entidad muy rara. Poster presented at: SECOT56 - Congreso de la Sociedad Española de Cirugía Ortopédica y Traumatología; 2019 Zaragoza, España España (2019). Extraña luxación de mediopie: disrupción de articulaciones escafocuneana y calcáneocuboidea, una entidad muy rara. https://congreso2019.secot.es/visor_posters_php.php?accio=download&id=5464.

[16] Salvi AE, Metelli GP, Domeneghini E, Florschutz AV, Bettinsoli R (2010). Diagnostic imaging and unforeseen associated lesions in astragalo-scaphoid dislocation: a case report. Arch Orthop Trauma Surg.

[17] Pérez RL, Pérez AL, Marco FL, Gilabert JE (2010). Exceptional midfoot dislocation: isolated dislocation of the calcaneocuboid joint. [Article in Spanish]. Rev Pie Tobillo.

